# Commodification anxiety and the memory of Turkish revolutionary Deniz Gezmiş

**DOI:** 10.1177/17506980241277517

**Published:** 2024-10-09

**Authors:** Duygu Erbil

**Affiliations:** Utrecht University, the Netherlands

**Keywords:** anti-capitalism, capital, celebrity culture, Marxism, memory-activism nexus, neoliberalism

## Abstract

This article examines the impact of commodification on the memory-activism nexus in relation to the cultural afterlife of Deniz Gezmiş. It reframes discussions of the ‘commodification’ of the revolutionary in terms of ‘celebrification’ and examines why this process generates social unease in Turkey. It shows that this anxiety emerges from the perception that once memory is brought into the circuit of exchange-value, it risks losing its use-value in activism. Cultural memory is indeed becoming increasingly mediated by market relations. Yet, this article calls attention to activist remembrance which occurs within the interstices of capitalist property relations and is therefore not necessarily *dependent* on the market. As such, it supports a shift from the ‘passive consumer’ paradigm to the recognition of the political and narrative agency of remembering subjects, demonstrating that people often contest processes of commodification, especially in the context of anti-capitalist activism.

The October 1998 issue of the Turkish monthly movie magazine *negatif* featured the Spanish actor Antonio Banderas on its cover, in celebration of the release of *The Mask of Zorro*. However, besides a spread of a topless Banderas wielding two guns and a handful of images emphasising the cleavages of other ‘sex symbols’ like Salma Hayek, there was little coverage of the ‘cover boy’ or the movie. Rather, the featured article was about the Ottoman Sultan Abdulhamid II, whose authoritarian reign left a contested legacy in Turkey. The title on the cover page, ‘Red Sultan or Great Khagan?’ [*Kızıl Sultan mı, Ulu Hakan mı?*], positioned the film magazine as a mediator in mnemonic contestations in Turkey. Among several other story titles adorning the two sides of the cover, there sat another enigmatic coverline, placed directly beneath the swashbuckling Banderas: ‘Is Deniz Gezmiş being exploited?’ [*Deniz Gezmiş sömürülüyor mu?*].

In 1998, the name Deniz Gezmiş needed no introduction, no more than the glamorous movie star or the notorious Ottoman sultan. The long-dead Marxist-Leninist revolutionary became the iconic symbol of Turkey’s 1968 movement following his execution by hanging in 1972. He was executed alongside two other comrades, Hüseyin İnan and Yusuf Aslan, as part of a larger group of student activists and militants who had died during those years of military crackdown. Yet it was Deniz Gezmiş who gained the reputation of ‘Turkey’s Che Guevara’. During his lifetime, Gezmiş had been the mediatic leader of the student movement, and in the decades following his death mainstream media increasingly individualised him as *the* icon of leftist politics in Turkey. If we take Chris Rojek’s (2001) definition of celebrity as the ‘attribution of glamorous or notorious status to an individual within the public sphere’ (p. 10), then, Gezmiş has become a historical celebrity; a glamorous role model for his fans and followers as well as a notorious symbol of dissent for those threatened by the social and political revolution as implied by his ongoing presence in Turkish public life. Decades after his death, even minor details about his life remain newsworthy within Turkey. Unearthing ‘unpublished’ photographs of Gezmiş is considered an event of mass public interest (Erbil, 2023: 79), as are recollections about his romantic entanglements and ‘declarations of love’ [*ilan-ı aşkları*] (Milliyet, 1998a). In other words, his fame has served the media industry well, as his name and image continue to function as a versatile attention generator. His name now serves as a keyword to guide online shoppers, with secondhand booksellers including Deniz Gezmiş in the product title of the October 1998 issue of *negatif*, which has become one of many collectables – ranging from old newspapers to coasters and t-shirts – that are adorned with his image. Add to that the broader media industry, which still repeatedly puts out Deniz Gezmiş stories to capture audience attention, which are subsequently sold to advertisers. In the years after 1998, the question posed by *negatif* has thus become increasingly pertinent for many: Is Deniz Gezmiş being exploited? Or, as sociologist Şehriban Şahin Kaya suggested, has he been simply reduced to one of many ‘commodities of remembrance’ [*hatırlama metaları*] (Şahin Kaya, 2011: 115)?

This question and the anxiety it reflects is of course not unique to Gezmiş. His status as the ‘Turkish Che Guevara’ directs us to the most prominent debates regarding the discomfort produced when the memories of anti-capitalist revolutionaries become entangled with the capitalist market. As Michael Casey (2009) notes, in writing about the ‘multimillion-dollar worldwide Che industry’ (p. 10), which produces branded products ranging from the infamous t-shirts to Hollywood films to condoms: ‘It’s impossible to overlook the irony . . . the commoditization of an anticapitalist rebel who opposed all that his hyper-commercialised image now represents’ (p. 30). Central to the affective and intellectual problematic posed by this ‘irony’ is an anxiety regarding the mnemonic effects of ‘commodification’, or ‘the power of the market to shape collective memories’ (Larson and Lizardo, 2007: 425). As Jeff A. Larson and Lizardo (2007) pose their enquiry into the afterlife of the South American revolutionary: ‘Has the image of Che Guevara lost its power to evoke radical politics in the face of pervasive commodification?’ (p. 425). This is the same problematic evoked in *negatif’s* provocative coverline regarding the potential ‘exploitation’ of Gezmiş. Yet, what often remains unclear in these discussions is the meaning attributed to ‘commodification’, evident in the frequent terminological slippage between the mere circulation of commodities that mediate memory and the full-fledged process of commodification. Delineating between the two thus may help us to better grasp the effects exerted on memory production and activism by the economic compulsions of capitalism.

This article examines why the production of the memory of activism under capitalism generates social unease, and how that unease plays into the dynamics of cultural remembrance as exhibited by the catchy coverline ‘Is Deniz Gezmiş being exploited?’. In this sense, I explore how *anxiety* about ‘commodification’ affects the memory-activism nexus; that is, the dynamic and co-constitutive relationship between the cultural remembrance of past activism, the uses of memory in activism and memory activism, which refers to activist endeavours to change collective memory (Rigney, 2018). I suggest that commercial uses of Gezmiş’s story and image, which I read as his ‘celebrification’ (Driessens, 2013: 643) rather than as absolute commodification, provoke anxiety in two ways. First, commercial use is taken to signify the contamination of the memory of the life and death of the anti-capitalist revolutionary, particularly for those who maintain a personal attachment to Gezmiş. The second variant of anxiety arises from the sense that the profitability of his image signals the depoliticisation of the memory of activism; once radical memories are brought into the circuit of exchange-value, they risk losing their use-value *in* anti-capitalist activism. The incorporation of counter-hegemonic memory into the circuit of capital appears to trigger an irreversible and vicious cycle, whereby the more public attention accrued by Gezmiş’s name and image, the more profitable he becomes for the very system against which he fought and which cost him his life. For some, capitalism appears to emerge victorious as they continue to struggle in vain against the appropriation of Gezmiş’s afterlife.

Nevertheless, contestations against the commodities that mediate cultural memory continue. While these contestations often assume a moralistic, non-materialist position against what is deemed the ‘commodification of memory’, they often reinvigorate a decommodifying process in the realm of cultural memory, specifically by opening and occupying spaces to remember outside market forces. In this essay, I highlight that the commercial uses of the past, like the use of Gezmiş’s image to sell t-shirts, does not constitute commodification *per se*. I take commodification to mean the subsumption of particular spheres of social life to market dependence, whereby they are subjected to the imperatives of accumulation, competition and profit-maximisation (Wood, 2013: 279). I suggest that the circulation of mnemonic commodities – commercial uses of the past – should not in itself be understood as constituting the commodification of memory and thus the neutralisation of its political potential. While the imagery and symbolism of radical politics may be appropriated by commercial actors, cultural remembrance itself often occurs within the interstices of capitalist property relations. Therefore, cultural memory is not (yet) entirely dependent upon the market for its practices and forms of reproduction. In this article, I seek to observe how contestations over mnemonic commodities challenge market imperatives and repoliticise the memory of anti-capitalist activism.

## The commodification of memory

The 1998 coverline of the movie magazine *negatif*, ‘Is Deniz Gezmiş being exploited?’, captured (or aimed to ignite) a sentiment that pertains to the perceived moral threat of money degrading or corrupting the inherent value of the thing it is exchanged for. This sentiment has been described by Vida Panitch (2024) as the ‘ontological corruption’ argument of philosophical anti-commodificationism (PAC) (pp. 74–75). According to this line of argumentation ‘things’ have inherent values, which make them unique and nonidentical, making exchanges incommensurable and leading to the ‘degrading of the goods themselves’ (Panitch, 2024: 74). In other words, money is taken to corrupt meaning once it enters the sacralised realms of social relations; the economic valuation of certain things causes moral injury. Melissa Hardesty (2019) calls this moral injury ‘commodification anxiety – the fear that intimate and sentimental relationships will be tainted or corrupted by the presence of money’ (p. 173). The commercial uses of the memory of activism generates exactly this type of anxiety, especially in the remembrance of anti-capitalist activism. I suggest that from a materialist perspective, this is a misplaced anxiety that arises from the intensified subsumption of life under the compulsions of the market. And from a cultural memory studies perspective, there is no need to fear the ‘ontological corruption’ of the cultural, political and social value of these figures of remembrance, as the field shows how their value is produced in active and intersubjective processes of remembrance, rather than resides in them, preceding their memory’s ‘exploitation’. Yet, still, the very existence of commodification anxiety invites an inquiry into the relation of markets to memory, and the meaning of commodification in this specific context.

In memory studies, the term ‘commodification’ is often employed to refer to the reification of the past as it is embodied in market commodities (see, e.g. Özyürek, 2004, 2007). However, this poses several definitional issues, since this use of ‘commodification’ may refer to myriad phenomena; from kitsch objects sold in museum shops and fashion accessories bought from online sellers, to commercial uses of the sentiments attached to traditional practices (Hart, 2007), and mass cultural artefacts (Landsberg, 2004). As Derek Hall (2023) points out, ‘commodification-of-everything’ claims are now omnipresent among leftist academics and activists alike, yet the definitions of ‘commodity’, ‘everything’ and ‘thing’ remain unclear (pp. 545–546). This lack of specificity is also evident when we look at claims about the ‘commodification of memory’. Despite the familiar sentiment that the profit motive contaminates cultural memory, especially when it relates to political potentialities, it remains unclear exactly how commodification affects memory.

To overcome this problem, Alison Landsberg (2004) coined the term ‘prosthetic memory’ to refer to cultural memories mediated through mass cultural commodities, observing that these ‘commodified memories’ trigger knee-jerk condemnations by intellectuals for reproducing ‘certain ideologies’ embedded in marketing strategies (p. 143). In response to the widespread assumption that ‘commodified memories’ pose an obstacle to collective action (Landsberg, 2004: 142), Landsberg (2004) suggested that ‘commodities as agents of social meaning are much less predictable than once assumed’ (p. 143), especially in the realm of culture where the passive consumer paradigm has been shifted by the recognition of consumer agency (pp. 143–144). Landsberg (2004) further emphasised the subversive nature of prosthetic memories, which circulate as mass cultural commodities:

As memories that no one person can own, that people can only share with others and whose meanings can never be completely stabilized, prosthetic memories themselves become a challenge to the ‘total possession’ of private property, by subverting the capitalist logic that produced them. (p. 147)

Arguing against the pessimistic takes on mnemonic commodities, Landsberg (2004) suggested that prosthetic memories ‘can never be owned as private property, and as a result they occupy a unique position within and yet implicitly opposed to capitalism’ (p. 147). Two decades later, Landsberg’s hopeful intervention against commodification anxiety remains relevant. However, much has changed in the last 20 years, starting with the monopolisation of the Internet, which Landsberg could still view as a potential democratising force in the production of prosthetic memories back in 2004 (p. 154). Recent calls for an examination of the ‘economy of memory’ and how memory reproduces capitalism (Palacios González, 2023) now urge us to take a step beyond resolving the problem of commodification through the recognition of consumer agency.

When we assign the meaning of commodification to the use of certain images or symbols in capitalist commodity production, almost all cultural memory under capitalism appears to be always-already commodified by definition. In this context, Landsberg’s (2004) urge to ‘use these commodified memories toward politically progressive ends’ (p. 146) remains an important reminder that consumers may choose to employ ‘products’ for political purposes. Nonetheless, addressing the problem of commodification at the point of consumption makes it difficult to start a conversation about memory-market relations, or about how the ‘economic power of capital’ (Mau, 2023) may shape remembrance practices alongside many other social relations. In this article, I agree with Landsberg in challenging the ‘passive consumer’ model. However, I also suggest that further specifying the dialectic between ‘commodification’ and ‘memory’ can contribute to the belated exploration of materialist perspectives in memory studies and to our understanding of the economy of memory. There are real threats to the production of cultural memory, posed by the monopolisation of communication technologies and, for example, intellectual property rights, which enable the trademarking of historical symbols. It is thus important to delineate between the mere market circulation of mnemonic commodities and the intensified commodification of mnemonic *practices*.

The assumption underlying the notion of a ‘commodified memory’ is the understanding of cultural or collective memory as a transactional system, whereby memories circulate as *goods*. In this framework, the transaction of memory belongs to what Margaret Jane Radin (2001) has called a ‘metaphorical market’, which frames social interactions as market transactions (p. 1). The ‘commodification of memory’, in this paradigm, resonates with Radin’s (2001) definition of ‘commodification’ as the *merging* of literal and metaphorical markets (p. 2); the economic valuation and transaction of market goods merging with the valuation and transaction of memories. This elision of metaphorical and literal markets in memory leads memory to become a struggle over ‘contested commodities’, or ‘controversies about what things can properly be bought and sold’ (Radin, 2001: xi). Memory-market relations, in this view, are confined to the *moral* question of whether it is appropriate for ‘memory’ to be brought into the circuit of exchange-value through the production of mnemonic commodities. This echoes the concerns of commodification studies, which searches for the moral limits of the market that would leave enough room for ‘human flourishing’ within an already existing market society (Panitch and Bertrand, 2024).

Speaking of memory in transactional terms, such as the ‘transmission’, ‘sharing’ and ‘inheritance’ of memory (e.g. Pickering and Keightley, 2013), indeed makes it appear as a ‘social good’ like love or kinship whose commodification may therefore be contested (Panitch, 2024: 73). But there is a fundamental problem with this equation of mnemonic commodities with the commodification of memory: that is, the pitfalls of conceptualising memory as a reified ‘thing’, referring to ‘THE collective memory of a society as ONE thing’ (Olick, 2007: 10, emphasis in original). Jeffrey Olick (2007) notably introduced the ‘process-relational’ concept of memory, foregrounding the role of ‘mnemonic practices’ as opposed to ‘THE memory as an entity’ (p. 10, emphasis in original). While we can speak of cultural ‘goods’, such as books and films, speaking of cultural memory as a good becomes problematic once one adopts this process-relational conceptualisation of memory. While the production of cultural goods mediates cultural memory by circulating certain representations of the past, processes of remembrance cannot be reduced to the exchange of these goods. As Olick (2007) maintains, mnemonic practices are *active* processes of remembrance ‘shaped by distinct constellations of forces, intentions, fantasies, and resources’ (p. 10), among which capital figures both as a force and a resource.

I want to suggest that in this dynamic model of cultural remembrance, the question of commodification should pertain to the question of those spheres of social life in which mnemonic practices are carried out by individuals and communities, rather than designate the mere existence of memory objects that are bought and sold within the capitalist market. Whereas capital figures as a resource in commodity production, it turns into a delimiting force in the case of commodification, constraining remembrance practices at the point of production: the increasing privatisation of the means of communication, or turning the symbols of the past legally into one’s private property to control their reproduction, constitute the subsumption of memory to capital. While the affirmation of consumer agency makes commodity circulation an irrelevant factor in remembrance practices, it lacks a solution to the problem of commodification that targets relations and processes rather than ‘things’. For the political Marxist Ellen Meiksins Wood, ‘commodification’ refers to the process by which a particular sphere of social life or activity becomes ‘market-dependent’, at which point actors, individually or collectively, are ‘forced to respond to the market’s imperatives’ of accumulation, competition, and profit-maximisation (Wood, 2013: 279). In this sense, rather than signifying the mere production of memory ‘goods’ that can be bought or sold on the capitalist market, the commodification of memory would entail the articulation of mnemonic practices to *market dependence*.

Given that memory is re/produced in multiple spheres of social life, it makes little sense to claim that all remembrance practices are subject to market dependence. However, this does not make the markets in memory irrelevant. As I will discuss below, commodification is an active force in memory cultures under capitalism, which activists resist, not only by decoding the ‘commodified memories’ differently, but by contesting the commodification process in various ways to achieve decommodification – the removal of sites and practices of remembrance from the domination of capital, denoting their democratisation. After all, as Søren Mau (2023) puts it, ‘the logic of capital, no matter how omnipotent it may seem, is only *one* social force among many’ (p. 293, emphasis in original). Taking this position allows us to avoid political despair while alerting us to the importance of identifying and mapping those sites of counter-hegemonic remembrance that are indeed threatened by the ‘direct control of capital’ and the ‘impersonal control of market-imperatives, which subordinate every human need and practice to the requirements of accumulation and profit-maximisation’ (Wood, 2013: 281). Centring the question of ‘commodification’ draws attention to the *production* of memory – how memory cultures are actively organised, structured and reproduced by individuals and communities – rather than forces us to think only in terms of the circulation and distribution of anti-capitalist histories and imaginaries as abstract processes involving reified ‘things’. I will now attempt to trace and delineate the interaction of some of these processes through a discussion of the commodification anxiety surrounding the afterlife of Deniz Gezmiş within Turkey. In doing so, I attempt to dispel some of the confusion that arises from the commercial uses of Gezmiş’s celebrity and its effects on forms of activist remembrance. This allows us to think more productively about the interaction of anti-capitalist activist memory and the capitalist market more broadly.

## From hero to celebrity

Concerns about the commodification and depoliticisation of Gezmiş’s memory must be contextualised within a Turkish left that witnessed the disintegration of civil society following the 1980 coup d’etat. The 1980 coup marked a complete socio-political rupture in Turkey and enforced a regime of forgetting as the junta aimed to ‘destroy the past itself’ (Zürcher, 2004: 279). The military junta of 1980–83 enacted an unprecedented repression of contentious politics and disintegrated the civil society that had come into being after the introduction of the 1961 constitution, which had democratised the political environment. It was in this environment that the 1968 movement flourished before escalating into a ‘civil war’ between the revolutionary left and the far-right, which was suppressed by the 1980 coup (Yenen, 2019). The coup reinforced political amnesia so as to erase the mobilising potential of the memories of past activism and accomplished a substantial depoliticisation of the public sphere. Hence, the anxiety regarding the depoliticisation of Gezmiş’s name and image has roots in this long history of persecution.

The military junta’s restrictions on the material and discursive opportunities for politicising memory work were not limited to the brutal repression of contentious politics and censorship, as the coup paved ‘the way in Chilean fashion for neoliberalization’ (Tuğal, 2021: 26). The gradual liberalisation of the Turkish economy from the 1980s onwards significantly impacted cultural production and accelerated fears that the capitalist market increasingly dominated the mediation of the past into cultural memory. The 1990s witnessed a partial liberalisation of the press, and official narratives began to be questioned more publicly, marking a ‘narrative opening’ for political stories to be told (Orhon, 2015: 66; Pekesen, 2020: 491). However, not *all* stories could be told, and the mass media infrastructure was now increasingly constrained by the dictates of the market. Here we can see the commodification of memory at play: an increasingly privatised media sphere, oriented exclusively towards the imperatives of profit-making and distanced from any form of democratic participation, controlled the means by which the most prominent narratives of the Turkish past could be disseminated. One of the key points to make here is that this process of de-democratisation is not to be confused with a mere autocratic or dictatorial control of the mass media of the sort that characterised the 1980–1983 junta, in which a military ruling bloc dictated the content of mass media. Rather, what marks the increasing privatisation of the media as commodification is the fact that the undemocratic character of media production was rooted in the market imperatives of competition, accumulation and profit-maximisation, homogenising media discourse through an emergent ‘oligopolistic ownership structure’ (Gür in Alpay, 2010: 276; see also Adaklı, 2009). The neoliberal restructuring of Turkey led to new ways of relating to the defeated revolutionary past in a commercial media environment that monetised everything, including the emotions the revolutionary times still evoked in the repressed left. In this way, the perceived depoliticisation of memory was a result of censorship and commodification working in tandem after the coup.

Before 1980, Deniz Gezmiş was understood to be a revolutionary martyr (Erbil, 2022), thus he was widely viewed as a *hero* whose self-sacrifice was a moral resource of legitimation and identification for Turkey’s *revolutionaries* (Yenen, 2019). After the wholesale persecution of the socialist revolutionary movement, and within the newly restructured media economy, Gezmiş all of a sudden began to figure as a famous historical actor in mass media representations, increasingly disarticulated from the revolutionary discourse of martyrdom. In other words, he became a historical celebrity and it was this process that focused anxieties regarding the commodification of his remembrance. In Daniel Boorstin’s (1992) classic definition, celebrity is the ‘human pseudo-event’ (p. 57); that is, a media fabrication of ‘human greatness’ (p. 58); a person ‘known for his well-knownness’ (p. 57), who replaced the ‘hero’, who ‘was distinguished by his achievement’ (p. 61): ‘The hero was a big man; the celebrity is a big name’ (p. 61). It is this distinction between the hero and the celebrity that often marks the anxiety surrounding the memory of Deniz Gezmiş in Turkey, who was a hero for many, but upon the privatisation of the Turkish mass media increasingly began to appear as a celebrity.

In the late 1980s, one significant change under the emergent neoliberal regime was the celebritisation of politics (and culture at large); that is, the process by which ‘celebrity, as an institution, diversifies and migrates, becoming an obvious organizing force’ in the political field (Elliott and Boyd, 2018: 12). This was due to the prevalence of tabloid journalism that undertook the task of political reportage within the restructured media economy to navigate political censorship and maximise profit. The 1980s witnessed the emergence of a ‘speaking Turkey’ that spoke in the register of gossip and lifestyle magazines (Gürbilek, 2001), and which signified the diversification of the celebrity framework and its application to the framing of political developments and current affairs. As celebritisation began to impact a range of social domains in Turkey alongside the commodification of mass media, Deniz Gezmiş’s name became increasingly prominent as companies attempted to capitalise on the public attention that he still generated within Turkish culture. For instance, the news magazine *Nokta*, which introduced agenda-setting sensationalist journalism to Turkey and became the highest circulating weekly by 1989 (Çeler, 2011; Sözeri, 2007), began to place Deniz Gezmiş and his iconic parka on its covers and special news (e.g. Nokta, 1987a, 1987b, 1988, 1990a, 1990b). He began to be treated as a celebrity.

After the introduction of commercial television broadcasting in Turkey in 1992, the story of Deniz Gezmiş was broadcast for the first time on the television channel Show TV in 1994, in a ten-part series produced by the *32. Gün* team *12 March: Democracy Under the Threat of Junta* [*12 Mart: İhtilalin Pençesinde Demokrasi*]. *12 March* instigated a year of discussion in newspapers. In her analysis of the documentary, Burçe Çelik (2020) counts in the daily *Milliyet* alone 60 news stories about Deniz Gezmiş between January 1994 and 1995 and suggests that the documentary contributed to ‘the popularization of the activists as “celebrities”’ (p. 59). Indeed, the popularity of the TV documentary and its ‘personalization of history’ (Çelik, 2020: 54) was instrumental in the celebrification of Deniz Gezmiş and his comrades. However, there was no public panic about Gezmiş’s celebrity until his story was fictionalised in Reis Çelik’s 1998 film *Goodbye Tomorrow [Hoşçakal Yarın]* and commercial media started to stir this sensational controversy.

## Misrepresenting Deniz Gezmiş

The 1998 debates around the supposed ‘exploitation’ of Gezmiş referred to by *negatif* was a reaction to the first cinematic representation of the revolutionary in Çelik’s film *Goodbye Tomorrow* [*Hoşçakal Yarın*] – and they began before the release of the film. The controversy around the film involved numerous social actors ranging from Gezmiş’s father to his comrades. Even Gezmiş’s former lover Avniye Anadol was asked to comment on the making of the film (Milliyet, 1998b). Newspapers and magazines seized on the sensational story. The group that most openly criticised the film, however, was the Foundation of the Union of 68ers [68’liler Birliği Vakfı], which was founded in 1992, or as they proclaimed, the ‘Year of Deniz Gezmiş’ (Yalçıner, 1993). They had their own controversial history, having previously been accused of profiting from the ‘68er’ identity and the memory of Gezmiş (Yalçıner, 1993). Indeed, ‘using’ Deniz Gezmiş had by now become a sensitive topic, but the first ever feature film about Gezmiş introduced new tensions.

Misrepresentation was the main source of the conflict. According to the Foundation representatives, Reis Çelik had sought their guidance on the film, leading them to form an advisory committee to help the director with the historical accuracy of Gezmiş’s story. However, according to them, Çelik had ignored their corrections and had deliberately misrepresented Gezmiş and the 1968 movement (Aygündüz, 1998). Their main critique was that Gezmiş appeared as an isolated man who was detached from the mass student movement and that the actor who played him had failed as terribly as the director because he had looked ‘very stressed and stuck’, as they asserted: ‘Deniz was a loving and warm man. It was impossible not to smile when you saw him’ (Atahan in Aygündüz, 1998: 51). Haşmet Atahan, who spoke as the president of the Foundation to *negatif*, was not the only person who thought his beloved Gezmiş had been stripped of his charm and charisma. Gezmiş was taken to represent the entire 1968 movement, but who was going to represent Gezmiş?

Even more importantly for others, Gezmiş’s politics were misrepresented in the film. Gezmiş’s primary school classmate and later comrade Aydın Çubukçu protested that his socialism was not covered at all in the movie (in Odabaş, 2003: 572). For Mustafa Yalçıner, who was a member of the guerrilla organisation People’s Liberation Army of Turkey co-founded by Gezmiş, workers, peasants and students constituted unacceptable absences from the film (in Odabaş, 2003: 572). Another critic of the film was Ertuğrul Kürkçü, a member of another revolutionary organisation during that period, which had taken three North Atlantic Treaty Organization’s (NATO) staff hostage in 1972 in an attempt to prevent the executions of Deniz Gezmiş and his comrades. Kürkçü was the sole survivor of the event, which came to be known as the ‘Kızıldere massacre’ as the military raid ended with the deaths of all the kidnappers and hostages except him. For Kürkçü, the main defect of the film was the omission of Gezmiş’s last words, where he venerates Marxism-Leninism (Kürkçü in Odabaş, 2023: 571). For all of these critics, the politics of the film did not represent the real politics of Gezmiş and those who revolted beside him.

We can see that this widespread disapproval of the film was initially motivated by anxiety regarding the intergenerational transmission of historical knowledge. The 68ers were concerned that the uninitiated youth, who lacked personal memories of the depicted historical events, would be misguided by the film’s representations. The youth might even become disillusioned with Deniz Gezmiş and the 1968 movement, they felt, because the story was simply that of ‘a man [being] caught in the field, taken away and hanged’ (Eşrefoğlu in Aygündüz, 1998: 51). This fear was also expressed by Deniz’s father Cemil Gezmiş, who dismissed the film as exploitative. Thus, the verdict was clear for the 68ers:

It is a fact that the film is insufficient to carry the values of the ‘68 to the present day. Despite this, I think that if the film has a great impact on the public in line with expectations, and is successful in being watched and earns big money, what Uncle Cemil said will inevitably come true. (Atahan in Aygündüz, 1998: 51)

As the quote indicates, they connect their anxiety regarding the veracity of historical representation with the presence of ‘big money’. As a student leader, Gezmiş’s story was supposed to transmit the spirit of the 1968 struggle to new generations and anything less was a deformation resulting from the capitalist drive for profit-maximisation. According to the foundation member Metin Eşrefoğlu, a Deniz Gezmiş film had to be a collective project and only through a public competition could they have found the perfect actor to play him, but instead it was Reis Çelik who had ‘wanted to make himself a hero’ (Eşrefoğlu in Aygündüz, 1998: 51). As a result, fame and money, social and economic capital, were distorting the first filmic representation of Deniz Gezmiş. The Foundation of the Union of 68ers concluded that they had to initiate a new film project to reflect the real spirit of ’68 and that this had to be a collective, collaborative project involving themselves as well as the revolutionary public (Atahan in Aygündüz, 1998: 51). In doing so, they expressed a decommodifying reflex against what they deemed the commodification of memory, asserting a democratic power against a commercial cinema that was driven not by social or historical motivations, but simply by a desire for profit, a form of ‘arbitrary power lodged in the “economic” sphere’ (Wood, 2020: 68).

In opposition to the 68ers who protested the commercial *use* of their friend and comrade, Reis Çelik’s response was equally accusatory. For him, the foundation did not recognise his authorial control over the language of cinema and tried to prevent him, in an ‘undemocratic’ manner, from making *his* film about the common, shared past of Turkey (Baştürk, 1998). In the end, the dispute boiled down to who *owned* what, in terms of both the symbolic imaginary of Turkey’s contentious history and the economic capital to produce narratives that remembered that history. According to media scholar Battal Odabaş (2003), who provides an overview of the controversy, the film fulfilled its political duty as it sparked a public discussion about the political past. However, the public debate led to no consensus or resolution besides an enduring incapacity to make films about Gezmiş, with the first Deniz Gezmiş film, remaining, to this day, the only one.

The irony is that media conglomerates came out ahead as this debate was not limited to one magazine. The controversy was carried to commercial television screens too, transfixing audience attention, which was by default sold to advertisers. The Foundation of the Union of 68ers enjoyed much attention during controversies like this as well as on the anniversaries of the execution of the three revolutionaries, because they were seen as the ‘rightful owners’ of the memory of Gezmiş and the 1968 movement. But as the airtime or paper space they were allocated generated audience attention, they helped both the advertisers to reach a wider audience and the media conglomerates to increase profits. This leads to the question: Who was exploiting who in this media economy? The real threat posed by commodification, that is, the question over who owns the means of communication to narrate the past, was mystified in mass media framings of the dispute over the film, which foregrounded a moralistic framework. Yet, the contestations over the film and the broader controversy surrounding the subjection of Gezmiş’s life story to commercial representations ended up being so acrimonious that no film production house ever successfully completed another adaptation. The commodification anxiety stirred by the mass media served the reproduction of capital in this instance, but it also re-popularised anti-capitalist sentiments in the remembrance of Gezmiş.

## Branding Deniz Gezmiş

Not all commodification scares have been as convoluted as the who-makes-profit-at-whose-cost riddle of the Turkish media economy. On 16 September 2011, a mysterious entrepreneur submitted an application at the Turkish Patent Institute for a ‘Deniz Gezmiş Parka Mont’. It was an attempt to trademark Gezmiş’s iconic green parka. The parka had become one of the most iconic symbols of the revolutionary left since militants wore it to signal their political allegiance. But, as it came to be particularly identified with Deniz Gezmiş, gradually turned into an ‘ideogram’ (Şeşen, 2016: 105). The reasons for the association of the parka with Gezmiş are multiple, ranging from the iconicity of Gezmiş’s arrest photograph, in which he was captured wearing the parka (Erbil, 2023), to numerous stories that set out to solve the ‘mystery’ of its origins (for an overview, see Şener, 2022). According to the sociologist Şehriban Şahin Kaya (2011), the parka also became a ‘nostalgic object’ following the popularity of the television series *Remember, Darling* [*Hatırla Sevgili*], which aired between 2006 and 2008. Just like the ‘nostalgic soundtrack’ and other period clothing that the historical melodrama popularised, the ‘Deniz Gezmiş parka’ became highly in demand during those years (Şahin Kaya, 2011: 114–115).

According to the owner of the costume sponsor of *Remember, Darling*, people of all ages from all over the country called to enquire about the outfits featured in the TV series (Vardar in Şahin Kaya, 2011: 115). It popularised not only the Deniz Gezmiş parka, but also women’s fashion from the 1960s and 1970s, reflecting what Şahin Kaya (2011) has described as the ‘commodification of the past’ (p. 115). She suggests that the culture industry turned Gezmiş and his friends from ‘figures of memory’ into ‘commodities of remembrance’ (Şahin Kaya, 2011: 115). The ‘misuse of memory’ [*Belleğin kötü kullanımı*] in ‘television that turns everything into entertainment’, she suggests, neutralises the critical potential of memory and puts it to use in praise of the existing order [*düzenini övücü kullanım*] (Şahin Kaya, 2011: 115). Şahin Kaya’s pessimism is based on a scholarly observation. Nevertheless, many ordinary people, who are often seen as being susceptible to the beguiling forces of the cultural industry, seem to share her judgement.

In newspapers, even the renewed public interest in biographical narratives about Gezmiş was framed entirely as a ‘boom in sales’ (Şahin Kaya, 2011: 114). One newspaper article emphasised that the buyers of these books were mostly female students of university and high school age (in Şahin Kaya, 2011: 114), suggestive of ‘girls’ buying books about Gezmiş, just as they desire to own dresses worn on television. A similar distrust of the ‘young generation’ was cultivated in a piece of anniversary journalism that asked people on the street if they knew Deniz Gezmiş, Hüseyin İnan and Yusuf Aslan. The title read ‘Even those who watched *Remember, Darling* do not know Deniz, Nobody knows Hüseyin and Yusuf’ (Özcan, 2008). While some academic and journalistic takes on *Remember, Darling* implied that the television series pacified audiences and stripped the past of all political potential, Nur Sarpkaya’s (2020) reception research has shown that audiences are not as apolitical and passive as this discourse claimed. She interviewed ten propertyless housewives without university education – arguably representative of a television soap audience that would be deemed passive from an elitist cultural framework. Her interviewees in fact displayed a very active engagement with the show: viewers criticised the reduction of left-right conflict to interpersonal issues and recognised the mystification of class struggle in the show. One interviewee claimed that she began watching the series to learn about Gezmiş (Sarpkaya, 2020: 222), while another condemned the show for ignoring the presence of American imperialism, which Gezmiş died fighting against (p. 232). Another interviewee pointed out that the show only represented ‘idealist and rich people’, whereas the leftist youth from the era were poor and working-class. One secondary school graduate bemoaned the fact that ‘nobody talks about equality’ in the show, and ‘everybody accepts the poor-rich division’ (Sarpkaya, 2020: 235). Sarpkaya (2020) concludes that although television encodes a universe of hegemonic ideology, this is always threatened and challenged by alternative decodings. Hence, she suggests, it would be a sign of real naivety to assume that people believe everything that television says and shows, as made evident by the interviewees’ responses to the television series (Sarpkaya, 2020: 255). This is a trenchant demonstration of the fact that mere *consumption* cannot be confused with the real process of commodification that restricts memory production. More importantly, these consumers retain the agency to challenge commodification, which was observable in the attempt to brand Deniz Gezmiş.

As the news broke about the entrepreneurial attempt to trademark the Deniz Gezmiş parka, ordinary Internet users started to raise their voices against this commodification attempt. For instance, in the collaborative hypertext dictionary, which has been allowing Turkish users to practice free speech online and produce and disseminate information or opinions about any topic since 1999, some users linked the entry [ ‘deniz gezmiş marka parka mont’] to familiar anti-capitalist critiques. One user cross-referenced the Marx quote ‘capitalism will cut down the tree if it can’t sell its shadow’, while another drew from the Communist Manifesto in lamenting that ‘all that is solid melts into air’ (Ekşi Sözlük, 2012). Another user cross-referenced the phrase ‘erosion of values’. These references demonstrated a clear recognition of the dangers posed by the commodification of Gezmiş’s memory; in this case it was not simply a matter of competing entrepreneurs employing the image of someone like Gezmiş or Che to sell t-shirts, but rather the attempt by a capitalist to turn the symbol of Gezmiş’s parka into his own private property through legal means in order to capitalise on its importance within the Turkish cultural imaginary. The hypertextual references on Ekşi Sözlük show that when social actors indeed perceive an ‘erosion of values’, that is, a shameless detachment of revolutionary memory from revolutionary politics in such a way as to render it *unusable* by other cultural actors, the response is not mere acceptance or weary resignation. The particular quotes from Marx cross-referenced by users of Ekşi Sözlük demonstrate that they were not responding simply to the cynicism of employing historical memory for profit-making, but to the threat that property relations pose to the re/production of memory itself. They recognised what Ellen Meiksins Wood (2020) has called the ‘arbitrary power’ lodged in the realm of productive relations: ‘Democracy requires, at the very least, that our liberties be protected by checking the “freedom” of the economy just as we check the “freedom” of the state’ (p. 68).

Moreover, the discursive work of contesting commodification worked in tandem with direct material action. On 19 February 2012, *Miliyet* asked Gezmiş’s older brother Bora Gezmiş to comment on the trademarking attempt, to which Bora Gezmiş replied that commercial uses of Deniz’s name transgressed the revolutionary’s political beliefs, and announced that he could trademark ‘Deniz Gezmiş’ himself in order to prevent attempts to commodify the revolutionary’s afterlife. At the time, Bora Gezmiş was also engaged in a dispute with the Ulucanlar Prison Museum (Milliyet, 2012), where Gezmiş, Aslan and İnan had been executed, and many Turkish public figures had served sentences. Ulucanlar Prison Museum opened in 2011 and began to sell cheap souvenirs such as lighters, t-shirts and mugs with Gezmiş and his comrades’ prints on them in the museum’s not-so-spectacular gift shop. Bora Gezmiş did not approve of this commercial enterprise even though the owner of the museum, Altındağ Municipality, insisted that they were not making a profit but merely meeting the demands of the museumgoers (Milliyet, 2012). However, the municipality closed the museum gift shop temporarily upon hearing about the dispute (Habertürk, 2012). The trademarking attempt failed too, affirming clear limits to entrepreneurial liberties. Yet the Deniz Gezmiş parka itself remained an iconic garment. Testament to this is how on 10 December 2013, the newspaper *Hürriyet* reported how Gezmiş was the ‘new victim of commercial brands that tried every way to promote their products’ since the ‘Deniz Gezmiş style parka’ was now a trending product title in e-commerce (Hürriyet, 2013), where the parka still circulated as a mnemonic commodity.

Despite the mainstream media rousing commodification anxiety, the collective knowledge-production project, 100 Years 100 Objects [*100 Sene 100 Nesne*], later presented a more optimistic perspective on the ongoing prominence of the parka. This project, which tells an alternate history of the Turkish Republic through a hundred objects, includes ‘Parka’ among its mnemonic objects. The author of the entry, Mustafa Şener (2022), uses the entry to tell the story of Gezmiş and the parka’s symbolic significance in leftist political culture, including the commodification and commodity circulation disputes sketched above. Yet, adding a hopeful note, he also connects this mnemonic object to the Gezi Park protests, which was a response to ‘authoritarian neoliberal urbanism’ crystallised by the attempted commodification of the park that belonged to the ‘urban commons’ (Kuymulu, 2013), by including the Gezi graffiti: ‘We are the park-going children of the parka-wearing generation’ [*parka giyen kuşağın parka giden çocuklarıyız*]. He also adds that some bought Deniz Gezmiş-style parkas to wear at the Gezi Park, connecting the memory of the Marxist-Leninist martyr with the civil uprisings in 2013, thus showing that public uses of the parka exceeded mindless consumerism. As such, the parka belonged to decommodification activism and its experimentation with ‘commoning – i.e. the social practices performed by commoners to manage and reclaim the commons’ (Varvarousis et al., 2021: 293). Şener’s framing is an affirmation that mnemonic commodities ‘may be used in unexpected ways that actively challenge the exploitative drive of capitalism’ (Landsberg, 2004: 152). But it is also ‘activist memory work’ (Merrill et al., 2020: 3) that repoliticises the parka, making it less of a commodity and more a political symbol available for the ‘park-going children’ (Şener, 2022).

Mustafa Şener’s story about the parka also includes the ongoing Rojava resistance for Kurdish liberation in Northern Syria that started in 2012, as he adds the story of Alican Vural to his assemblage of parka memories. Şener tells us that the 17-year-old Vural posted a photograph of himself trying on a Deniz Gezmiş parka he saw in an expensive store in 2013, with the caption: ‘This is the revolutionary spirit, taking a photo in the store as a souvenir when you cannot afford the parka you dream of’. Two years later, Vural was killed during the 2015 Suruç bombing, when a suicide attack targeted a group of student activists who were giving a press statement of solidarity with the Rojava resistance. Vural’s earlier post with the Deniz Gezmiş parka resonated with many as it captured the endurance of a youthful revolutionary spirit instead of mindless consumerism (Aksakal, 2016). In Şener’s narrative of the parka, Alican Vural hence found a place among other memories of resistance. His act of solidarity was memorialised through the decommodifying reflex of activist memory work that attributed political meaning to his desire to own a Deniz Gezmiş parka, which, within more pessimistic frameworks, might simply have been dismissed as a young man falling victim to consumer culture. As Mustafa Şener shows, however, not all buyers of the Deniz Gezmiş parka are ‘victims’ of a consumer culture. Indeed, many like Alican Vural retain the motivation to self-represent as revolutionaries when they buy or express a desire to own a ‘Deniz Gezmiş Parka’. The purchase is not of a reified memory *per se*, but an act of identification with Gezmiş and his politics, and an act of identifying oneself as a leftist to the world. In this sense, the phenomenon of the ‘Deniz Gezmiş parka’ in the consumer market can be better explained as an outcome of a celebrity culture than as the commodification of the revolutionary symbolism, as reflected in the trademark attempt.

The commodification anxiety regarding the celebrification of Deniz Gezmiş may seem irrelevant since cultural remembrance includes agents who actively engage with mnemonic commodities, even when they are produced for passive consumption. Celebrity scholars also show us that celebrity culture involves more than the reduction of people to images and, more importantly, that ‘fans’ are neither passive consumers nor mere victims of commercial culture (Elliott and Boyd, 2018; Stevenson, 2018). This echoes Landsberg’s (2004) affirmation that commodities can be used in unexpected ways and hence do not pose an inherent danger to collective action. But what is more interesting than whether or not mnemonic commodities have political potential is how ‘consumers’ contest the process of commodification. These contestations effectively turn cultural memory into a site of contesting capitalism by creating cultural commons and decommodifying the means to remember.

## Conclusion

Cultural memory is a terrain of ongoing struggle, as the abundant scholarship on mnemonic contestations has shown us. What I aimed to show is that these contestations also include contesting markets in memory and the commodification of memory at large. This article began by questioning what we mean by the ‘commodification of memory’, which causes much anxiety when it comes to the remembrance of anti-capitalist pasts within a capitalist world order. I demonstrated that commodification is often taken to refer to the way that certain images and symbols are ‘sold’ as products under capitalism, which leads to a moral assessment of whether a ‘commodified memory’ is a ‘contested commodity’ or not, and if so, whether we may remedy this moral injury through affirming consumer agency. Throughout the case study, I offered some concrete examples to show that this affirmative attitude can in fact be supported by evidence once we turn to the intentional agents of cultural remembrance and politics, instead of focusing solely on reified memories produced as commodities. Indeed, the cultural remembrance of anti-capitalist struggles from the past continues to inspire new activists, who mobilise these memories in anti-capitalist activism and relate to these historical figures in myriad ways. However, I also argued that focusing solely on consumer agency in relation to mnemonic commodities provides only a limited perspective on memory-market relations, and it remains necessary to examine the threat posed by commodification itself, which radically delimits human agency by making social practices market-dependent.

In this article, I argued for a delineation between the circulation of mnemonic commodities and the process of commodification to avoid losing sight of the coercive power of capital over mnemonic practices when we affirm the agency of the remembering subjects who sit at the consumption end of memory production. More systematic research into the ‘economy of memory’ (Palacios González, 2023) that takes into account property relations ranging from media ownership structures to intellectual property laws legislating copyright, patents and trademarks will surely shed more light on what I have situated as the commodification of memory. Pending that, this essay aimed to show that many people recognise the threat of commodification and contest these enforced property relations that delimit cultural remembrance practices.

I also showed that what we often call the ‘commodification’ of a historical figure can be more accurately described as celebrification, which does not denote complete market dependency. The commercial uses of Gezmiş’s name and image can be understood as celebrification as the term brings together both the uses of his infamous story in the Turkish cultural industry and the ways in which his fame is used to sell other market commodities. Celebrification indeed complicates Gezmiş’s remembrance as it makes him more usable for the mass media industry and other capitalist entrepreneurs. Both the active/participatory and passive engagement of fans with celebrified historical figures like Deniz Gezmiş or Che Guevara, make an interesting topic for future discussions. The blurring of the lines between political alliance and fandom will have to consider the ‘celebrity activism nexus’ proposed by Red Chidgey (2021), and this relationship’s consequences for the ‘memory-activism nexus’ (Rigney, 2018). The relationship between celebrities and social movements is often conflicted. Chidgey (2021) suggests that since ‘celebrity is the very embodiment of a marketable commodity’ and celebrity culture legitimates capitalist models of exchange and value, celebrity activism is often taken to be an ‘oxymoron’ in feminist media scholarship (p. 1056). As she points out: ‘[w]ithin this scholarship, celebrity is routinely seen as media-friendly, commodified, and premised on individualism, rather than an activist need for collectivist politics’ (Chidgey, 2021: 1056). This assumption, as we observed, also marks the pushback against the celebrification of Gezmiş, because if his afterlife is reduced to his commercial image, and if his remembrance becomes a fandom practice, what is left of his politics or the uses of his memory in activism? This is why the decommodification of his memory entails an emphasis on his place in the socialist movement.

On one hand, it is important to recognise that the increasing commodification of mnemonic practices, through the increasing privatisation of the means of communication within Turkey, threatens and indeed limits the possibilities for radical remembrances of Gezmiş to circulate on a mass scale. On the other hand, the celebrification of Gezmiş has not resulted in the complete depoliticisation of cultural memory given that memory is always contested and that cultural remembrance is not reducible to the question of ‘mass communications’ and the ownership of its specific technologies. In examining the pervasive anxiety that attends to the intersection of Gezmiş’s memory with the capitalist market in Turkey, I have argued for the relevance of distinguishing between mnemonic commodities that draw on the celebrity of the anti-capitalist revolutionary and the commodification of memory, as the process by which spheres of social life in which anti-capitalist memory cultures flourish are tied to market dependency. While Deniz Gezmiş’s face may adorn t-shirts and lighters that circulate in the market, it is also hoisted by protestors on banners, spray-painted onto buildings and printed on political leaflets (Erbil, 2023). The sites and cultures in which his memory and its meaning are produced and contested often reside outside of market relations and cannot be accurately captured by pessimistic accounts of the ‘commodification of everything’. An important task is to identify the threats posed by commodification to these remembrance practices, especially since many of these activists, in the spirit of Gezmiş himself, mobilise his memory in activism with the aim of decommodifying life itself.
